# Insulin resistance and fertility in polycystic ovary syndrome.

**Published:** 2008-11-15

**Authors:** S Fica, A Albu, M Constantin, GA Dobri

**Affiliations:** * ‘Carol Davila’ University of Medicine and Pharmacy, Bucharest, Endocrinology Department , Romania; ** ‘Elias’ Hospital, Bucharest, Endocrinology, Diabetes and Metabolism DepartmentRomania

## Abstract

Polycystic Ovary Syndrome (PCOS) represents a common endocrinopathy, with anovulation and hyperandrogenism as cardinal symptoms. In recent years it has been recognized that insulin resistance is an intrinsec feature of the disorder and plays a central role in pathogenesis. PCOS is associated with important reproductive morbidity as shown by high prevalence of anovulatory infertility, spontaneous abortion, gestational diabetes and pre–eclampsia. The association of insulin resistance with this reproductive pathology has been well documented. Due to major implication of insulin resistance in PCOS pathogenesis, insulin reduction strategies were studied as a possible treatment for infertility in PCOS patients. Weight loss, even modest was proved to be a simple and efficient method to improve reproductive parameters in PCOS patients and should be recommended to all overweight and obese patients with infertility. Metformin was showed to induce ovulation, at least in a subset of patients with PCOS, but there are not unequivocal proves concerning its efficacy for pregnancies and live–birth rate, mainly because few trials studied this aspect. Therefore there are not enough evidences to recommend metformin for infertility treatment in PCOS. Few small studies with newer thiazolidindiones suggest their efficacy for ovulation induction, but further extensive studies are needed to confirm these results. In conclusion, reduction of insulin resistance was proved to ameliorate ovulation rate in PCOS patients, but strong evidences to sustain the utility of insulin–sensitizing drugs as a therapeutic option for infertility are lacking. Future studies are needed to elucidate these aspects and to characterize the particular subtype of patients with higher probability to respond to this treatment.

## Introduction

PCOS is one of the most frequent endocrinopathies in the reproductive age years, with a prevalence of 5–10%[1,2]. At the same time, PCOS is the most frequent cause of hyperandrogenism and anovulatory related infertility. Although it was initially described by Stein an Leventhal in 1935, as a reproductive disorder characterized by polycystic ovaries, menstrual abnormalities, infertility and obesity, the studies of the last thirty years have demonstrated that PCOS is a disorder associated with an increased risk of type 2 diabetes mellitus, metabolic syndrome and cardiovascular disease. Nowadays it is believed that insulin resistance is the cause for this increased risk. Insulin resistance is a common finding in PCOS patients, with an important role in its pathogenesis. Considering that insulin resistance and secondary hyperinsulinemia are involved in the occurrence of clinical and paraclinical features of PCOS, many authors have studied the efficiency of insulin resistance reduction strategies in the PCOS treatment.

## PCOS Diagnosis

The criteria used for PCOS diagnosis have changed in time. Until 2003, the PCOS diagnosis was based on criteria established by the National Institute of Health (NIH) Conference in 1990: clinical and/or biochemical hyperandrogenism associated with chronic anovulation. Exclusion of other ovarian, adrenal, thyroid and pituitary disorders are necessary for supporting the diagnosis. In 2003, the International Conference held in Rotterdam includes the polycystic aspect of the ovaries on transvaginal ultrasound as a diagnostic criterion along with hyperandrogenism and oligo–anovulation, recommending that PCOS diagnosis should be established if two out of the three criteria were present. Thus, polycystic ovaries are considered a diagnostic criterion, although some authors consider this not to be specific [3] because it can also be found in patients without hyperandrogenism and anovulation [3,4]. After the Rotterdam criteria were introduced, two new PCOS phenotypes have been described besides the classic one: patients with polycystic ovaries, hyperandrogenism and ovulatory cycles (ovulatory phenotype), and patients with polycystic ovaries and anovulation, but without hyperandrogenism. Studying these subtypes of patients, it has been observed that patients with the ovulatory phenotype have less insulin resistance than the classic phenotype (5), and those without hyperandrogenism have no insulin resistance [6]. In these subtypes the involvement of insulin resistance in the pathogenesis may be less important. This is why the use of Rotterdam criteria may produce confusions when patients other then belonging to the classic phenotype are introduced in clinical studies referring to insulin resistance or metabolic abnormalities. Taking in consideration these aspects, Androgen Excess Society (AES) defines PCOS as a disorder in which androgen excess is defining and recommends to be included in PCOS category only patients with hyperandrogenism. The data available till now about the involvement of insulin resistance and high risk for metabolic abnormalities derive from studies that have included mainly patients with the classic phenotype.

## Insulin resistance in PCOS

The association between insulin resistance and hyperandrogenism has been reported for the first time in 1921, when Achard and Thiers have described ‘diabetes des femmes a barbe’ [7]. Nowadays, the relationship between the two conditions is well documented, especialy based on studies showing that PCOS patients have insulin resistance and compensatory hyperinsulinemia. Although insulin resistance is considered an intrinsec feature of the syndrome, independent of body weight, obesity, a frequent feature [8] among PCOS patients (60–70%), has a major contribution to the aggravation of the insulin resistance. Insulin resistance was also reported in lean PCOS patients, but its prevalence was variable and some studies even failed to find it. Nowadays, PCOS is considered a disease with a complex and heterogeneous pathogenesis, different mechanisms being involved in different ratios in the appearance of the syndrome. Therefore in certain patients, insulin resistance may be less involved in the pathogenesis.

The etiology of insulin resistance in PCOS, although intensely studied, is not completely cleared up. The mechanisms most probably involved are defects at the post receptor level. Thus, in 50% of the patients autophosphorylation of the insulin receptor (IR), which inhibits the intrinsic tyrosine–kinase activity of the IR, has been described [9]. Another post receptor anomaly has been described in the muscle cells of the PCOS patients. This consists in the decrease of the insulin–mediated phosphatidylinositol–3–kinase activity associated to IRS1 (insulin receptor substrate 1), involved in the glucose tapping and carbohydrate metabolism (10). Regarding the existence of other IR anomalies, it is possible that a small number of IR in adipocytes to be involved in the insulin resistance of PCOS, the hypothesis being related in several studies. In exchange, the decrease of insulin affinity for its receptors is not considered to have major implications in PCOS pathogenesis.

It is well known that the abdominal and visceral adiposity is associated with insulin resistance. In PCOS patients an android disposition of adipose tissue has been described in the obese patients, as well as in the normal weight patients. Moreover, lean PCOS have much more adipose tissue than the normal weight controls. These anthropometric characteristics may contribute to the PCOS insulin resistance. The particular disposition of adipose tissue in PCOS could be explained by lipolysis anomalies similar to that found in the patients with android obesity and could be explained by a decreased density of beta2 adrenergic receptors and hormone–sensible lipase activity.

Reduced fetal growth could be another possible mechanism of PCOS insulin resistance. This has been demonstrated to be a risk factor for metabolic and cardiovascular complications in the adult life, presumably due to its association with insulin resistance. Furthermore, it has been shown that reduced fetal growth predisposes to hyperinsulinemia, anovulation and ovarian hyperandrogenism in patients with premature pubarche [11]. These data suggest that postpubertal appearance of the typical PCOS manifestations may be antenatal conditioned, with the possible contribution of the environmental and genetic factors.

The involvement of insulin resistance and secondary hyperinsulinemia in the PCOS pathogenesis is complex, implying the existence of many mechanisms. In vivo and in vitro studies have proved that insulin directly stimulates ovarian streroidogenesis at the level of theca cells, along with LH. This co–gonadotropic effect contributes to the PCOS hyperandrogenism. Furthermore insulin decreases the sex hormone binding globulin (SHBG) levels by decreasing the liver synthesis, raising the level of free testosterone. Also, it decreases the IGFBP1 liver synthesis and so it increases IGF1 biodisponibility in the ovaries, causing increased production of ovarian androgens through its co–gonadotropic effect. In 50% of the PCOS patients, the adrenal androgens have an important contribution in the appearance of hyperandrogenism, the involvement of hyperinsulinemia in the alteration of the adrenal steroid genesis being demonstrated [12]. Although experimental studies suggested the contribution of hyperinsulinemia to the characteristic changes of gonadotrop secretion in PCOS, the clinical studies didn't succeed to offer solid evidence [13].

Recently, it has been demonstrated that insulin action in the ovaries is mediated by inositolglycan at the post receptor level, and not by tyrosine–kinase cascade. This is how we explain why the insulin resistance, through IR autophosphorylation, in other tissues doesn't impede the insulin effects in the ovaries [14].

These mechanisms through which insulin resistance contributes to the appearance of clinical and paraclinical changes in PCOS are mostly demonstrated by in vitro studies. In vivo studies have tried to reproduce these mechanisms by acute administration of insulin, but they obtained contradictory results. Of the clinical studies, the most convincing have been those that proved the amelioration of the clinical and paraclinical parameters by decreasing the insulin resistance, thus supporting its pathogenic role.

## Infertility in PCOS

Infertility is frequently met in PCOS patients, with a prevalence of 74%[15], and in 40% of cases it is actually the motive of presentation to the doctor[16]. Infertility is the consequence of chronic anovulation, being usually accompanied by menstrual abnormalities: dysfunctional uterine bleeding, oligomenhorrea, amenorrhea. The presence of regular menstrual bleeding doesn't exclude anovulation. Twenty one per cent of the patients with hyperandrogenism have regular menstrual cycles that are anovulatory [17]. Furthermore PCOS patients that get pregnant spontaneously or after ovulation induction treatments have more frequently pregnancy associated pathology. So, these patients have an increased risk of spontaneous abortion. The exact mechanism that would explain this complication is still a matter of debate, both hyperandrogenism and obesity being incriminated as major causes. There are studies that show an increased prevalence of gestational diabetes (40–46%) [18] and pregnancy induced hypertension (28,5%) [19] and have proved the association between insulin resistance and these pregnancy complications [18].

The exact mechanism that determines the chronic anovulation in PCOS has been deeply studied, but in spite of all the studies conducted till now, we don't have a complete understanding of all the components implicated in this process. The hystologic studies performed in the PCOS patients have shown ovarian follicles arrested in evolution in the antral stage, containing degenerate granulosa cells. Based on these initial studies it has been thought that the big number of ovarian follicles found in PCOS are atretic. But subsequent studies demonstrated that the PCOS follicles are viable and functional, preserving the capacity of steroidogenesis [20]. Furthermore the granulosa cells of PCOS patients have an increased sensibility to FSH stimulation, proving once more their viability. Although some authors affirm that the increased ovarian androgens level induces follicular atresia and incapacity of selection of a dominant follicle [21], there are numerous proves that contradict this hypothesis. Studies made on animals have shown that testosterone administration raises the number of normal preantral and antral follicles [22]; in these follicles testosterone increases the mARN of the androgenic receptors in the granulosa cells, determining their own proliferation and inhibition of apoptosis [23]. Furthermore testosterone increases the mARN of the FSH receptor in the granulosa cells [24]. All these support the role of androgens in increasing the number of small but functional follicles, pointing to hyperandrogenism as the main factor to induce the ovarian changes in PCOS.

The increased sensibility of the granulosa cells to FSH and the big number of viable follicles determine constant levels of estradiol and inhibin, with the loss of physiologic variations of the estradiol levels that permit the FSH and LH peak, leading to anovulation. Hyperinsulinemia indirectly contributes to anovulation, through hyperandrogenism, but may have a direct role, by stimulating the production of estradiol the same as FSH.

## Strategies of reducing insulin resistance in PCOS

Since there are numerous evidences of the involvement of insulin resistance in the PCOS pathogenesis, strategies of insulin resistance reduction have been studied in the treatment of this syndrome. The utility of insulin resistance reduction has been initially suggested by the studies that followed the effect of loosing weight in obese PCOS patients. Afterward, there were studies about the use of insulin sensitizing drugs in PCOS. First such a report was given in 1994, by Velazquez, who observed the decrease of the free androgens and an improvement of the menstrual abnormalities in the patients that have received Metformin [25]. In the majority of studies, the favorable effects on the endocrine and reproductive anomalies have been accompanied by the reduction of the insulin resistance, supporting its etiologic involvement.

***Weight loss*** has been studied by many authors, all of them reporting the improvement of hyperinsulinemia and hyperandrogenism, simultaneously with the recovery of normal menstrual cycles and the appearance of spontaneous pregnancies in 30% of the patients [26]. These fortunate effects were observed even with a modest weight loss of 2–5% [27]. Because of the association between obesity and increased risk for spontaneous abortion, the weight loss could have a positive effect on the outcome of the pregnancy, although this aspect hasn't been particularly studied in PCOS. In obese patients, weight loss is associated with the improvement of the lipid and cardiovascular parameters, with benefits in the long run. Also considering the good effects upon the fertility, weight loss must be recommended to all obese patients with PCOS as an adjoin to any chosen therapy.

***Metformin*** was the first insulin sensitizing drug to be given to the PCOS patients, initially with the purpose of obtaining additional arguments regarding the involvement of insulin resistance in the PCOS pathogenesis. Although the study conducted by Velasquez in 1994 proved the efficiency of Metformin in the improvement of the endocrine status at the same time with the insulin resistance reduction, the parallel weight loss of the patients could have been a source of error. But the subsequent studies have demonstrated that the Metformin effects are independent of the weight variations [28,29]. Administered to the PCOS patients, Metformin may improve the menstrual disorders and anovulation in 50% of the studied patients, with increased probability of response in the patients with hyperinsulinemia, less severe menstrual disorders and a lower level of free testosterone [28] . Thus, it has been speculated upon the fact that Metformin is efficient in the category of patients in which the insulin resistance has an important role in pathogenesis. Not all the studies have succeeded in proving the efficiency of Metformin in PCOS patients regarding the decrease of hyperinsulinemia and hyperandrogenism [30,31]. The explanation could be the severe obesity of the patients in those studies, being well known that an important weight loss is necessary to decrease the insulin resistance in severely obese patients. Because only 50% of the PCOS patients respond to Metformin, the proportion of these patients in the studied group could influence the final results. Furthermore, it has been proven that you have to give 2,550mg/day to lower the insulin resistance in the obese women, doses like 1,500 mg having no effect [32]. Nevertheless, most of the studies have used 1,500 to 1,700 mg/day.

Now, there are studies that prove that Metformin has positive results in the normoponderal patients with PCOS, showing a higher efficiency when comparing to the supraponderal and obese patients (33).

Clomiphene citrate is the classic therapy used in the induction of ovulation, being successful in 75% of the cases. The rest of 25% are considered resistant to Clomiphene and are usually more obese with a higher insulin resistance. The administration of Metformin before Clomiphene, in the PCOS patients, has shown a significant increase of ovulation and pregnancy rates when compared to placebo [34,35]. There is a well known association between insulin resistance and the risk of ovarian hyperstimulation syndrome; when Metformin is administered in association with the gonadotrops, a smaller number of follicles, lower levels of estradiol and higher number of fertilizations and pregnancies are observed [36].

In the PCOS patients that become pregnant there is a high risk of complications, like spontaneous abortion and gestational diabetes. Administered in pregnancy, it has been proved that Metformin lowers the risk of these complications. The effect may be due to the decrease in insulin resistance and hyperandrogenism, but also to other mechanisms: the normalization of the PAI activity and the modulation of the immune response of the endometrium (reflected by the high levels of glycodelin induced by Metformin) [37]. The safety of giving Metformin during pregnancy hasn't been proved, being categorized as B drug. Thus, the benefits of Metformin in pregnancy must be confirmed by extensive studies, after its safety has been proved.

Because most of the Metformin studies are small and have no control group, metaanalyses of the randomized controlled trials have been made. A recent metaanalysis (April 2008) that took in consideration 17 studies with a total of 1,639 patients confirmed the efficiency of Metformin in the ovulation induction, compared with placebo, especially in women without resistance to Clomiphene (38). At the same time, the association of Metformin and Clomiphene is superior to Clomiphene alone in ovulation induction and obtaining the pregnancy, especially in the obese women or resistant to Clomiphene (38). But to estimate the value of an infertility treatment the most important is the final result: the birth of a live neonate. Very few studies had as an objective this parameter. Recently two big, randomized studies that followed the efficiency of Metformin in increasing the number of live neonates have been published. The first study, done on approximately 100 normoponderal PCOS patients, observed a similar efficiency of Metformin and Clomiphene in inducing ovulation, but superior results in obtaining the pregnancy for Metformin [39]. The subsequent pursuit of these patients has shown a bigger number of live neonates in the group treated with Metformin. The second study comprised 600 patients (200 patients per each study group) and compared the efficiency of Metformin and Clomiphene administered separately and in association. Although a superior efficiency in ovulation induction has been observed in the patients that receives the combined treatment, this wasn't followed by a significantly bigger number of live neonates [40]. An explanation for the difference between the results of the two studies may be given by the high percentage of obese patients in the second study (aprox. 70%), being known the high risk of spontaneous abortion caused by the obesity alone. In these studies, the Metformin administration has been stopped the moment the pregnancy was obtained, so the possible effect of abortion reduction (reported in previous studies) that could have improved the outcome of the pregnancy could not be studied.

***Thiazolidindiones*** After clinical studies have demonstrated the efficiency of Metformin in the amelioration of the endocrine and reproductive parameters, it has been tried to study other insulin sensitizing drugs in the treatment of PCOS. In 2001, Azziz reported the results of a multicentre study on 410 PCOS patients (41) who received Troglitazone in doses of 150, 300 and 600mg/day for 44 weeks. It has been observed the improvement of the hyperandrogenism, hyperinsulinism, the menstrual abnormalities and anovulation in a dose–dependent manner. The patients that responded to the treatment were less obese, with less severe menstrual abnormalities, hyperinsulinism and hyperandrogenism. In 2000, Troglitazone was withdrawn from use due to the severe liver adverse reactions. Thus, the utility of the new thiazolidindiones (Rosiglitazone and Pioglitazone) is now evaluated in PCOS patients. Rosiglitazone in 4mg/day dose [42] or Pioglitazone 30mg/day dose [43] were proved to reduce the insulin resistance and hyperinsulinemia in PCOS patients, as well as restoring the ovulation and the regular menstrual cycles. In normoponderal PCOS patients, the clinical and paraclinical amelioration was obtained with 2 mg/day of Rosiglitazone, as well as with 4 mg/day, with a greater effect of the bigger dose [44]. Rosiglitazone, in doses of 4mg/day, determined clinical and paraclinical improvement in obese patients with increased insulin resistance considered less responsive to Metformin [45]. It was suggested that Rosiglitazone could represent an alternative to the treatment of obese patients with high insulin resistance when Metformin is not efficient. Similar studies have shown that Rosiglitazone (8mg/day), as well as Pioglitazone (30mg/day), have superior results in the amelioration of insulin resistance and hyperandrogenism, in the supraponderal and obese PCOS patients, when compared to Metformin (2,000–2,550 mg/day) [46,47]. Furthermore, in patients resistant to Clomiphene, the association with Rosiglitazone proved to have better outcome then Metformin association in ovulation induction, although there was no significant difference in the pregnancy number between the two groups [48]. Unlike Metformin that produces weight loss, thiazolidindiones don't have this favorable effect, but most studies have shown a decrease in the waist/hip ratio because of the fat redistribution process induced by thiazolidindiones, with possible modification of the insulin resistance. These arguments of the thiazolidindiones efficacy in the treatment of PCOS come from studies made with a small number of patients that is why they must be confirmed by bigger randomized studies. Relying on a metaanalysis, it has been recently suggested that Rosiglitazone might be associated with an increased risk of coronary events, but later studies didn't confirm this suspicion. It can't be overlooked that thiazolidindiones are placed in the C category regarding pregnancy, because animal studies have shown a delay in the fetal development. That is why when you intend ovulation induction, this drug administration must be made with precaution and stopped when pregnancy appears.

## Conclusions

Insulin resistance involvement in PCOS pathogenesis is clearly supported by in vivo and in vitro studies, the most convincing being those that involve insulin resistance reduction strategies. The insulin sensitizing drugs have induced in most of the studies, the improvement of the endocrine and reproductive parameters simultaneously with the amelioration of the insulin resistance and hyperinsulinemia, suggesting this to be the main beneficial mechanism in PCOS. Data available today support the efficacy of Metformin in ovulation induction, and its' association with Clomiphene increases the rate of ovulation and pregnancies in PCOS patients resistant to Clomiphene. The favorable effects of Metformin are not universal, but vary with PCOS subtype, patients with a higher insulin resistance having a better response. At the same time, presence of severe obesity may limit the efficacy of Metformin treatment. In these situations, the thiazolidindiones could be a therapeutic alternative. Considering all these data, there is controversy regarding the final aim represented by the increase in the number of live neonates, in part because of the small number of studies. While this effect was described in the normal weight patients, in the supraponderal and obese PCOS patients this effect was not reported. This fact may be due to the discontinuance of Metformin treatment at the beginning of the pregnancy and with this the possible effect of spontaneous abortion reduction. Weight loss associated with Metformin treatment might improve the pregnancy outcome and must be recommended to all supraponderal and obese PCOS patients. More studies are needed to establish exactly the categories of patients that respond to the insulin sensitizing drugs to increase the efficiency of the therapeutic strategies. Also, it must be evaluated the safety of Metformin use during pregnancy and the possible implications in the increase of the live neonates number.
